# Peripheral Signatures of Multidimensional Pathology in Symptomatic and Asymptomatic Creutzfeldt–Jakob Disease

**DOI:** 10.1002/cns.70765

**Published:** 2026-01-23

**Authors:** Zhong‐Yun Chen, Min Chu, Yi‐Hao Wang, Rui Liu, Jing Zhang, Ai‐ling Yue, Hua Lu, Qian‐qian He, Jia‐hui Hou, Yu‐fei Chen, Hong Ye, Li‐Yong Wu

**Affiliations:** ^1^ Department of Neurology, Xuanwu Hospital Capital Medical University Beijing China; ^2^ National Clinical Research Center for Geriatric Disorders, Xuanwu Hospital Capital Medical University Beijing China

**Keywords:** blood–brain barrier, Creutzfeldt–Jakob disease, genetic, prion, prognosis

## Abstract

**Background:**

Plasma markers of neuronal injury in Creutzfeldt–Jakob disease (CJD) are established, but peripheral biomarkers reflecting glial activation, synaptic dysfunction, and vascular impairment remain less explored. We systematically assessed these markers in symptomatic CJD and asymptomatic PRNP mutation carriers to improve diagnosis and identify early pathophysiology.

**Methods:**

This prospective cohort study recruited CJD, frontotemporal dementia (FTD), healthy controls (HCs), and preclinical familial CJD pedigrees. Sixteen plasma proteins representing neuronal injury, glial activation, synaptic function, and vascular/BBB integrity were measured. Analyses included group differences, discriminative performance, clinical/imaging correlations, and longitudinal trajectories.

**Results:**

We enrolled 130 CJD patients, 145 FTD, 70 HCs, 16 asymptomatic *PRNP* carriers (4–6 years follow‐up, 4 converters), and 16 non‐carrier family controls. In symptomatic CJD, plasma NfL, t‐tau, and GFAP were strongly elevated, each showing excellent discriminative performance (AUCs > 0.93 vs. HCs and > 0.82 vs. FTD). We also observed alterations in vascular/BBB markers, with VCAM‐1 levels elevated and significantly associated with both clinical decline and DWI hyperintensity. In asymptomatic carriers, biomarker levels remained largely normal and stable preclinically. Notably, two G114V carriers showed mild pre‐symptomatic elevations in NfL and GFAP, and one exhibited a slight VCAM‐1 increase before clinical onset; all changes exceeded the 90th percentile of control values. E200K and T188K carriers showed no pre‐onset changes.

**Conclusions:**

Plasma biomarkers in CJD may reflect multisystem involvement, with neuronal markers showing strong discriminative potential and vascular proteins indicating possible BBB dysfunction. In asymptomatic carriers, minor changes may occur only near onset in those with relatively slow‐progressing mutations.

## Graphical abstract

Plasma biomarkers in CJD reveal multisystem involvement, with neuronal injury markers showing strong discriminative performance and vascular proteins indicating blood–brain barrier dysfunction. In asymptomatic *PRNP* mutation carriers, biomarker changes are minimal and emerge mainly near disease onset

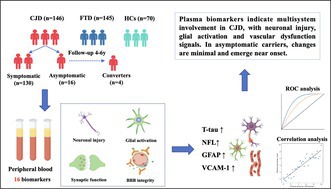

## 1 Introduction

Prion diseases are a group of fatal and rapidly progressive neurodegenerative disorders characterized by the misfolding of the normal cellular prion protein (PrP^C^) into its pathogenic isoform (PrP^Sc^). Among them, Creutzfeldt–Jakob disease (CJD) is the most common form in humans. While the accumulation of PrP^Sc^ remains a central hallmark of disease pathology, accumulating evidence from both animal models and human studies indicates that prion disease progression is not solely driven by protein misfolding. Instead, a cascade of secondary pathological processes—such as glial activation, neuroinflammation, synaptic dysfunction, and disruption of the blood–brain barrier (BBB)—appears to interact and collectively contribute to neuronal injury and clinical deterioration [1, 2, 3, 4].

Recent advances in fluid biomarker research have enabled the detection of molecular signatures reflecting such multifaceted pathophysiological changes across neurodegenerative diseases. In CJD, cerebrospinal fluid (CSF) markers like total tau (t‐tau) and neurofilament light chain (NfL) have shown robust diagnostic performance, and their plasma analogues are now recognized as promising, less invasive alternatives [5, 6, 7]. However, blood‐based biomarkers encompassing a wider array of pathological domains remain insufficiently explored in CJD. Although supported by preclinical and postmortem findings, it remains uncertain whether these diverse mechanisms are simultaneously active and detectable in the peripheral circulation of CJD patients [4, 8].

Importantly, previous research has largely concentrated on the symptomatic stages of CJD, while the molecular alterations that may precede clinical onset—especially in individuals with genetic mutations—remain poorly characterized [9, 10, 11, 12]. Understanding whether systemic biological changes can be detected during the preclinical phase is crucial not only for elucidating disease mechanisms but also for identifying early biomarkers that could guide future therapeutic interventions.

In this study, we comprehensively investigated a panel of plasma biomarkers representative of key pathological pathways implicated in CJD. By integrating biochemical data with clinical and imaging features, we aimed to identify systemic alterations across disease stages and assess their potential for early detection and longitudinal monitoring. By extending biomarker research beyond traditional CSF‐based markers to peripheral signatures, this study provides new insights into the complex and systemic pathophysiology of CJD.

## 2 Methods

### 2.1 Study Design and Participants

This study was conducted in compliance with the ethical principles set forth in the Declaration of Helsinki (1974) and its subsequent amendments. Approval was granted by the Ethics Committee of Xuanwu Hospital, Capital Medical University (Approval No. 2024‐180‐002). Written informed consent was obtained from all participating individuals and their families.

Participants were recruited from the Department of Neurology at Xuanwu Hospital, Capital Medical University, Beijing, China, between January 2019 and December 2024. A total of 130 symptomatic patients with CJD were included, comprising 119 individuals diagnosed with probable sporadic CJD (sCJD) based on the modified WHO criteria and 19 genetically confirmed cases of genetic CJD (gCJD), including E200K (*n* = 9), T188K (*n* = 5), G114V (*n* = 2), T193I (*n* = 1), R148H (*n* = 1) and E196K (*n* = 1) [13]. All symptomatic patients were followed every 3 months, in person or by telephone, until death to confirm diagnosis and monitor disease progression. Exclusion criteria for symptomatic CJD patients included refusal to participate, alternative diagnoses such as autoimmune encephalitis or paraneoplastic syndromes, or confirmation of other diagnoses during follow‐up.

In addition, 16 asymptomatic *PRNP* mutation carriers from four pedigrees (one G114V, two T188K, and one E200K family) were enrolled as the preclinical CJD group. These individuals were followed longitudinally for 4–6 years, during which four carriers developed symptomatic gCJD. Sixteen non‐carrier relatives from the same families were included as familial controls. Seventy healthy controls were recruited from the community, all with no history of cognitive, psychiatric, or neurological disorders, and normal cognitive performance (Mini‐Mental State Examination score ≥ 24). A total of 145 patients with frontotemporal dementia (FTD) were recruited as disease controls. The FTD group included individuals diagnosed with probable behavioral variant FTD (bvFTD) according to the revised international consensus criteria [14], or with semantic variant or nonfluent/agrammatic variant primary progressive aphasia according to the diagnostic framework proposed by Gorno‐Tempini and colleagues [15].

### 2.2 Clinical Screening

All participants underwent comprehensive clinical evaluations, including cognitive screening with the MMSE and Montreal Cognitive Assessment (MoCA). Disease severity was assessed using the Medical Research Council‐Prion Disease Rating Scale (MRC‐PDRS), a validated 20‐point functional scale (ranging from 0, indicating a comatose or vegetative state, to 20, reflecting full independence in activities of daily living) [16]. Disease duration was defined as the interval between symptom onset and death. The degree of disease progression is defined as the ratio of the interval between onset and sampling time to the total disease duration. The number of hyperintense regions on DWI was scored across the frontal, temporal, parietal, and occipital lobes, caudate nucleus, putamen, and thalamus (1 point per region; 2 points if bilaterally involved) [17]. Additional diagnostic evaluations included electroencephalography (EEG), cerebrospinal fluid 14‐3‐3 protein testing, real‐time quaking‐induced conversion (RT‐QuIC), and analysis of *PRNP* codon 129 polymorphisms.

### 2.3 Plasma Collection and Biomarker Profiling

Blood samples were drawn from fasting participants and collected in EDTA‐coated tubes (BD, USA). The entire procedure, from blood collection to processing, was conducted at room temperature. Plasma was obtained by centrifuging the samples at 3000 rpm for 10 min, then aliquoted and preserved at –80°C for future analysis.

Sixteen plasma proteins were selected to reflect four major pathological domains implicated in CJD. These included markers of glial activation and neuroinflammation: Galectin‐3, chitinase‐3‐like protein 1 (YKL‐40), soluble triggering receptor expressed on myeloid cells 2 (sTREM2), glial fibrillary acidic protein (GFAP), chitotriosidase (CHIT1), and chemokine (C‐X3‐C motif) ligand 1 (CX3CL1, also known as fractalkine); markers of vascular/blood–brain barrier (BBB) integrity: vascular cell adhesion molecule 1 (VCAM‐1), matrix metalloproteinase 9 (MMP‐9), vascular endothelial growth factor A (VEGF‐A), aquaporin‐4 (AQP4), transforming growth factor beta 1 (TGF‐β1), and platelet‐derived growth factor‐BB (PDGF‐BB); markers of neuronal/axonal injury: NfL and t‐tau; and markers of synaptic function and plasticity: neuronal pentraxin‐2 (NPTX2) and neuronal pentraxin receptor (NPTXR).

For biomarker analysis, various immunoassay platforms were used to ensure sensitive and accurate quantification. Luminex xMAP technology (R&D Systems) was employed for the measurement of Galectin‐3 (1.7 pg/mL sensitivity), MMP‐9 (13.6 pg/mL sensitivity), PDGF‐BB (0.2 pg/mL sensitivity), YKL‐40 (3.3 pg/mL sensitivity), CX3CL1 (64.8 pg/mL sensitivity), VCAM‐1 (238 pg/mL sensitivity), and VEGF‐A (0.99 pg/mL sensitivity). For electrochemiluminescence immunoassay, the S‐PLEX platform (Meso Scale Discovery) was used to quantify GFAP (150 fg/mL sensitivity), NfL (930 fg/mL sensitivity), and t‐tau (37 fg/mL sensitivity). ELISA methods were applied for the detection of TREM‐2 (28 pg/mL sensitivity), AQP‐4 (0.19 ng/mL sensitivity), TGF‐β1 (less than 9.3 pg/mL sensitivity), CHIT1 (8 pg/mL sensitivity), NPTXR (0.4 ng/mL sensitivity), and NPTX2 (0.094 ng/mL sensitivity).

All Luminex assays were performed at LabEx of UNIV (Shanghai, China) following manufacturer protocols. Samples were incubated with antibody‐coupled magnetic beads, followed by biotinylated detection antibodies and streptavidin‐phycoerythrin. Fluorescence signals were acquired using the Luminex X‐200 platform, and concentrations were calculated from standard curves using Milliplex Analyst Version 5.1. Each batch included quality controls to ensure data reliability and reduce assay variability; the intra‐ and inter‐plate coefficients of variation for all quantified proteins are provided in the Supporting Information.

### 2.4 Statistical Analysis

For group comparisons, different statistical approaches were applied depending on data distribution. Normality was assessed using the Shapiro–Wilk test. Variables following a normal distribution were compared using independent samples *t*‐tests, whereas non‐normally distributed variables were either log₁₀(x + 1)‐transformed prior to analysis or analyzed using the Mann–Whitney *U* test if normality was not achieved after transformation. Adjusted analyses were performed using linear regression models (y ~ group + age) to control for age effects. Categorical variables were compared using the chi‐square (*χ* ^2^) test. Multiple comparisons were corrected using the Benjamini–Hochberg false discovery rate (FDR) procedure. To evaluate the discriminative performance of plasma biomarkers, receiver operating characteristic (ROC) curve analyses were conducted. The area under the curve (AUC) was calculated to quantify overall discriminative ability, with 95% confidence intervals (CIs) used to indicate precision. The optimal cut‐off point was determined by the Youden index, maximizing the sum of sensitivity and specificity. Partial correlation analyses (adjusted for age, sex, and time from onset to sampling) were used for pairwise associations between plasma biomarkers and clinical/imaging measures. To further evaluate these associations, multivariable linear regression models were applied, adjusting for the same covariates. Multiple comparisons within each outcome domain were corrected using the Benjamini–Hochberg FDR method. All tests were two‐tailed, with a significance threshold of *p* < 0.05. Univariate and multivariate Cox regression analyses were then performed to assess the association between survival and continuous values of each biomarker. All statistical analyses and visualizations were conducted using R software (version 4.0.3; R Foundation for Statistical Computing, Vienna, Austria), GraphPad Prism 7.00 (GraphPad Software, San Diego, CA), and IBM SPSS Statistics 26 (IBM Corp., Armonk, NY).

## 3 Results

### 3.1 Demographics of the Participants

Table 1. presents the demographic and clinical characteristics of the patients with CJD, FTD, and HCs. Among the 130 CJD patients, 64 (49.2%) were male and 66 (50.8%) were female, with a mean age of 60.5 years. Most patients were in the early to middle stages of disease progression, with the interval from symptom onset to blood sampling representing a median of 28.5% of the total disease duration. The rates of positive diagnostic indicators were as follows: CSF 14‐3‐3 protein (61.1%), periodic sharp wave complexes on EEG (41.5%), hyperintensity on DWI (93.1%), and positive RT‐QuIC results in CSF or skin samples (94.7%). *PRNP* codon 129 genotype analyses revealed that 98.5% of patients were methionine homozygotes (129MM).

**TABLE 1:** Demographic and clinical features of patients with CJD, FTD and HCs.

Characteristic	CJD (*n* = 130)	FTD (*n* = 145)	HCs (*n* = 70)	*p* ^a^	*p* ^b^
Patient information					
Age at onset, year	60.5 ± 9.4	63.0 ± 8.7	62.7 ± 7.9	0.019	0.324
Sex, male, *n* (%)	64 (49.2)	55 (37.9)	32 (40.0)	0.059	0.271
Onset to sample, days, median (IQR)	73.5 (46.0, 104.3)	—	—	—	—
Disease progression^c^, %	28.5 (18.4, 48.1)	—	—	—	—
Duration, days, median (IQR)	258.0 (174.5, 367.0)	—	—	—	—
Clinical features at presentation, *n* (%)					
Cognitive	125 (96.2)	145 (100.0)	—	0.023	—
Psychiatric	74 (56.9)	45 (31.0)	—	< 0.001	—
Visual	46 (35.4)	16 (11.0)	—	< 0.001	—
Extrapyramidal	72 (55.4)	24 (16.6)	—	< 0.001	—
Pyramidal	65 (50.0)	12 (8.3)	—	< 0.001	—
Cerebellar	73 (56.2)	8 (6.5)	—	< 0.001	—
Myoclonus	46 (35.4)	0	—	< 0.001	—
Mutism	8 (6.2)	0	—	0.002	—
Scale evaluation					
MMSE, median (IQR)	6.0 (0, 18.5)	13.0 (10, 20.0)	28.5 (27.0,29.3)	< 0.001	< 0.001
MoCA, median (IQR)	3.0 (0, 10.0)	7.0 (4.0, 14.0)	25.0 (24.0, 27.0)	< 0.001	< 0.001
MRC‐PDRS, median (IQR)	12.0 (7.0, 17.0)	—	—	—	—
CSF 14‐3‐3 positive, *n* (%)	74/121 (61.1)	—	—	—	—
Periodic discharges on EEG, *n* (%)	54 (41.5)	—	—	—	—
Hyperintensity on DWI, *n* (%)	121 (93.1)	0	—	< 0.001	—
Number of involved lobes	7 (4.8, 8.0)	—	—	—	—
Positive RT‐QuIC, *n* (%)	89/94 (94.7)	—	—	—	—
Codon 129 MM genotype, *n* (%)	128 (98.5)	—	—	—	—

Abbreviations: CJD, Creutzfeldt‐Jakob disease; DWI, Diffusion Weighted Imaging; EEG, Electroencephalogram; FTD, frontotemporal dementia; HCs, Healthy controls; IQR, interquartile range; MM, methionine homozygosity; MMSE, mini‐mental state examination; MoCA, Montreal Cognitive Assessment; MRC‐PDRS, MRC Prion Disease Rating Scale; RT‐QuIC, Real‐time quaking‐induced conversion.

^a^ CJD vs. FTD.

^b^ CJD vs. HCs.

^c^ Defined as the ratio between the interval from onset to sampling and the total disease duration.

### 3.2 Altered Plasma Biomarker Profiles in Symptomatic CJD

**FIGURE 1:**
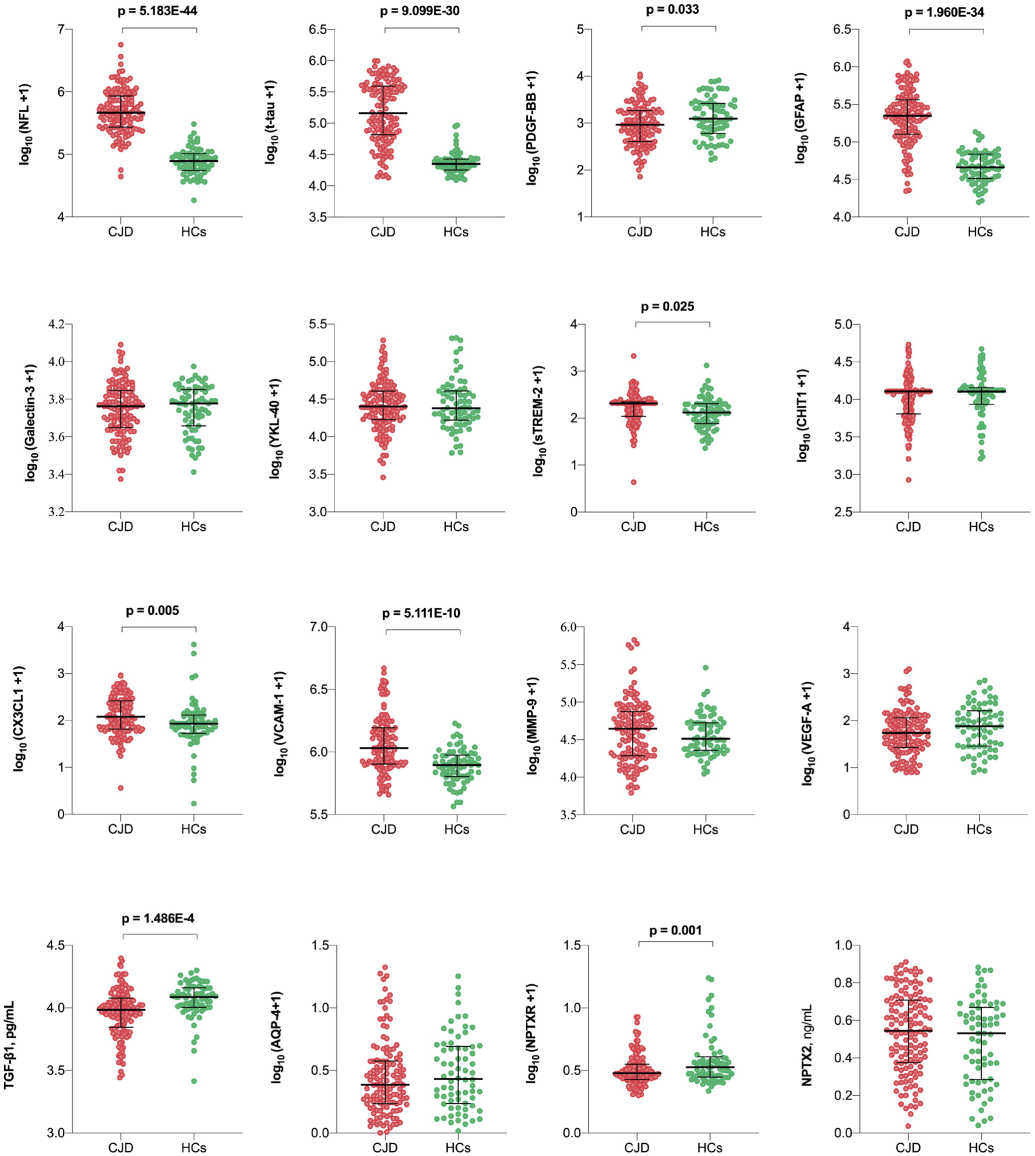
Plasma biomarker alterations in clinical CJD. Group comparisons of plasma biomarker levels between CJD and healthy control (HC) groups. Data were log_10_(*x* + 1)‐transformed and are presented as median±interquartile range.

As shown in Figure 1 and Table S1, symptomatic CJD patients exhibited pronounced alterations in plasma biomarker profiles across multiple pathological domains. Consistent with previous studies, markers of neuronal and astroglial injury—including NfL, t‐tau, and GFAP—were markedly elevated in CJD compared with healthy controls (all *p* < 0.001). Markers associated with BBB integrity also showed substantial changes. VCAM‐1 levels were significantly increased, while TGF‐β1 concentrations were markedly decreased; both remained highly significant after adjustment for age and FDR correction (both *p* < 0.001). In addition, neuroinflammatory mediators such as sTREM2 (*p* = 0.008) and CX3CL1 (*p* = 0.004) were elevated, whereas PDGF‐BB was reduced (*p* = 0.019). The synaptic marker NPTXR was significantly decreased compared with healthy controls (*p* = 0.013), and these differences remained robust after age adjustment and FDR correction.

When compared with FTD, CJD showed higher levels of GFAP, NfL, t‐tau, and VCAM‐1, and lower levels of MMP‐9 and CX3CL1 (Table S2). Differences in synaptic proteins were also observed, with decreased NPTXR and increased NPTX2 in CJD.

Comparisons of biomarker levels were also made between sCJD and gCJD. Among these, galectin‐3 was significantly lower in gCJD than in sCJD (*p* = 0.002), and the difference remained significant after adjustment for age (Table S3).

### 3.3 Inter‐Biomarker Correlations in Symptomatic CJD

**FIGURE 2:**
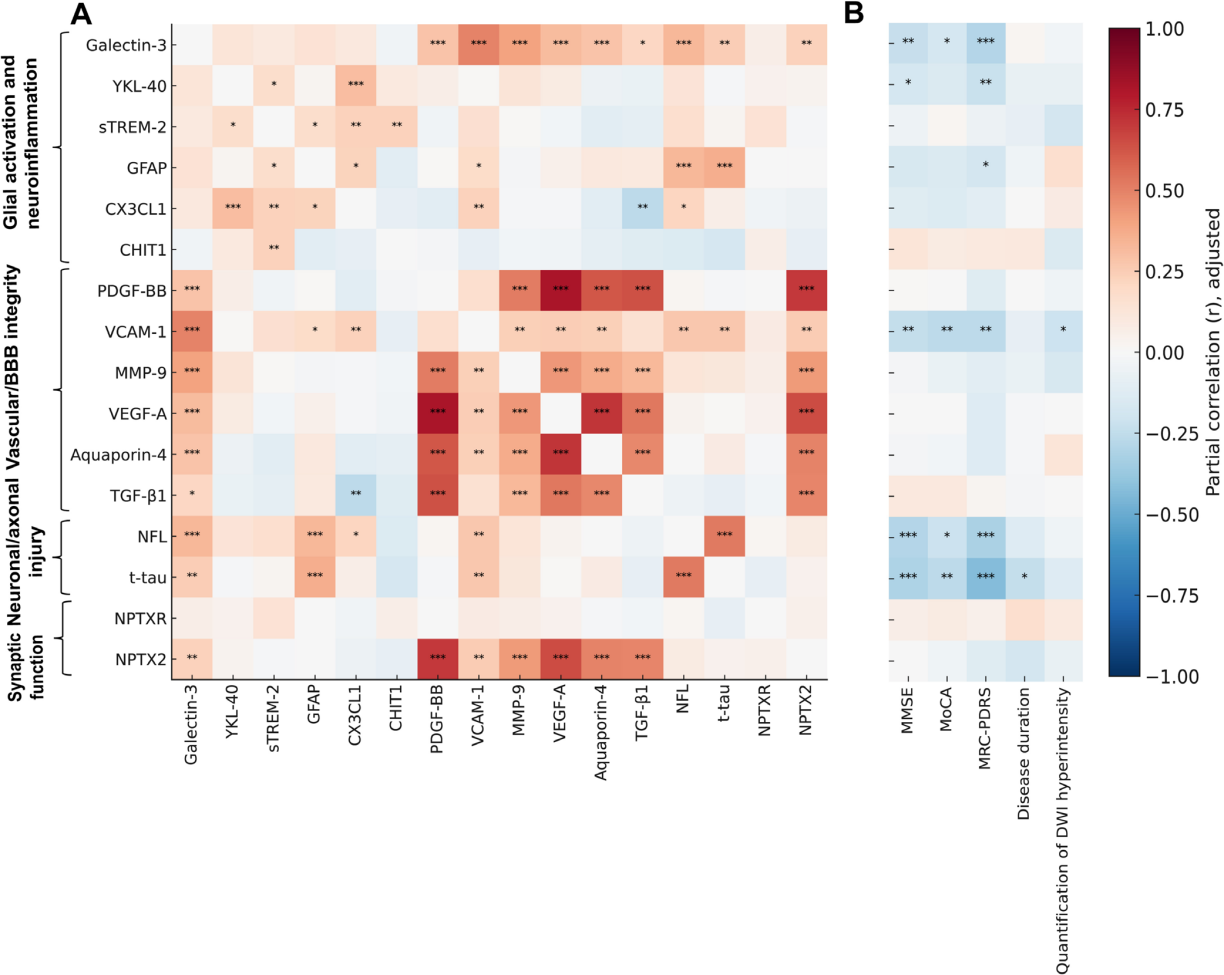
Correlation analysis of plasma biomarkers. (A) Partial correlation analysis among plasma biomarkers, adjusting for sex, age, and time from onset to sampling. (B) Partial correlation analysis between plasma biomarkers and clinical or imaging measures, adjusting for sex, age, and time to sample. Significance levels are indicated as *p < 0.05, **p < 0.01, ***p < 0.001.

As shown in Figure 2A, after adjustment for age, sex, and time from onset to sample, plasma biomarkers related to BBB integrity were significantly intercorrelated. Notably, NPTX2 and Galectin‐3 also showed strong associations with multiple BBB‐related markers, suggesting potential cross‐talk between synaptic dysfunction, glial activation, and vascular dysfunction. NfL and t‐tau, reflecting neuronal injury, were closely correlated with Galectin‐3 and GFAP, highlighting their linkage with glial responses. In addition, GFAP was associated with VCAM‐1, indicating a possible connection between astrocytic activation and BBB disruption.

### 3.4 Discriminative Utility of Plasma Biomarkers for CJD

**FIGURE 3:**
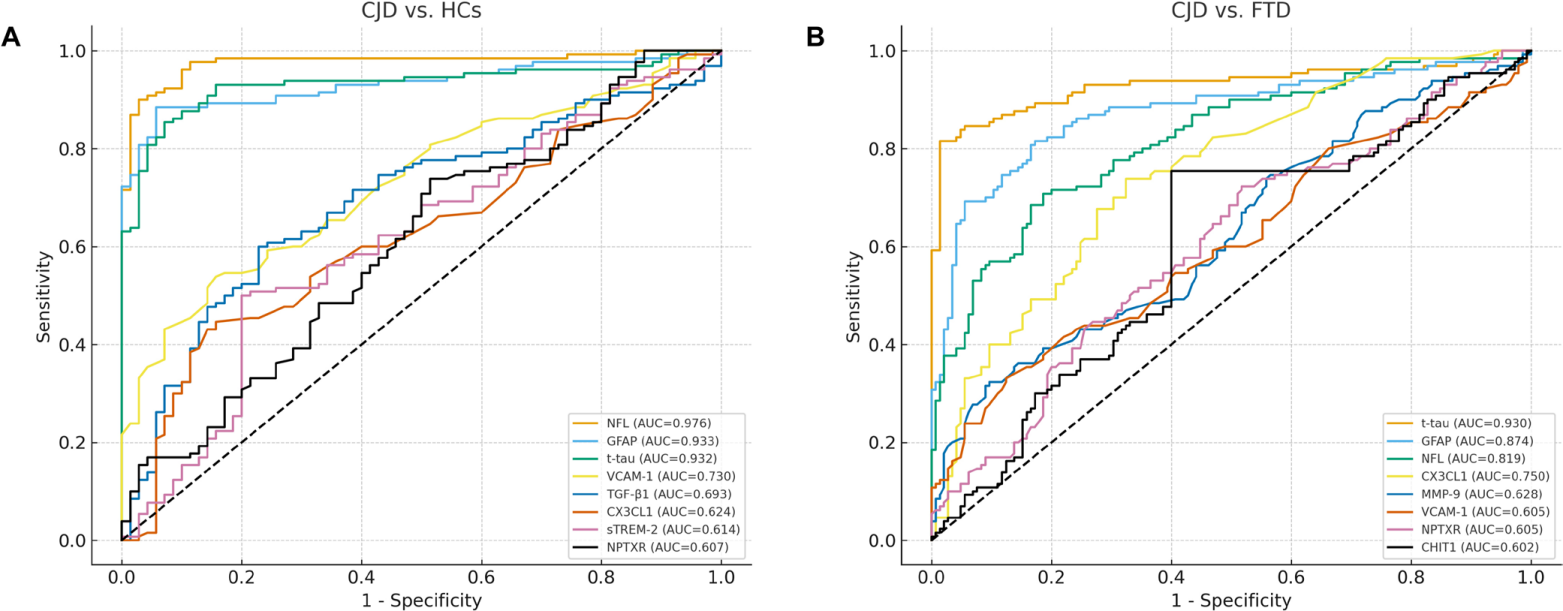
ROC analysis between CJD and control/FTD groups. Receiver operating characteristic curve analyses comparing plasma biomarkers between CJD and HC groups (A), and between CJD and FTD groups (B).

As shown in Figure 3A and Table S4, ROC analyses were performed to evaluate the group‐separating performance of plasma biomarkers to distinguish CJD patients from HCs. Among all biomarkers, NfL showed the highest discriminative performance, with an AUC of 0.976, followed by GFAP (AUC = 0.933) and t‐tau (AUC = 0.932). VCAM‐1 demonstrated a moderate discriminative ability (AUC = 0.730; sensitivity = 0.538; specificity = 0.843). In contrast, individual markers such as TGF‐β1, CX3CL1, PDGF‐BB, and NPTXR exhibited relatively low AUC values when used alone.

To further examine the differential discriminative potential in other neurodegenerative diseases, biomarker performance was also evaluated between CJD and FTD (Figure 3B and Table S5). Consistent with findings against HCs, t‐tau, NfL, and GFAP remained the most discriminative markers (AUCs = 0.930, 0.819, and 0.874, respectively). VCAM‐1 showed only limited accuracy (AUC = 0.605). Interestingly, CX3CL1 yielded an AUC of 0.750, suggesting a moderate ability to differentiate CJD from FTD.

### 3.5 Prognostic Implications of Biomarker Levels

After adjustment for age, sex, and time to sample, several plasma biomarkers demonstrated significant associations with cognitive and functional measures (Figure 2B, Table S6). Higher levels of t‐tau and NfL were strongly associated with lower MMSE (t‐tau: FDR *p* = 0.006; NfL: FDR p = 0.006) and MoCA (t‐tau: FDR *p* = 0.020) scores, both remaining significant after FDR correction. VCAM‐1 also showed significant negative correlations with both MMSE (FDR *p* = 0.029) and MoCA (FDR p = 0.020). Regarding disease severity, t‐tau, NfL, VCAM‐1, and Galectin‐3 were all negatively correlated with MRC‐PDRS scores; these associations remained significant after FDR correction (t‐tau: FDR *p* = 4.79 × 10^−6^; NfL: FDR *p* = 0.002; VCAM‐1: FDR *p* = 0.010; Galectin‐3: FDR *p* = 0.004). In addition, t‐tau showed a significant inverse association with disease duration (FDR *p* = 0.031). To further assess the prognostic relevance of plasma biomarkers, Cox proportional hazards regression analyses were performed with survival time as the outcome. After adjustment for age, sex, and time from onset to sampling, t‐tau emerged as the only biomarker significantly associated with survival, with higher t‐tau levels predicting shorter survival duration (HR = 1.729, 95% CI 1.102–2.712, *p* = 0.017). No other biomarkers showed significant associations with survival after covariate adjustment (Table S7). A nominal correlation was observed between VCAM‐1 and DWI hyperintensity quantification, but this did not survive correction for multiple comparisons.

### 3.6 Longitudinal Biomarker Dynamics in Preclinical gCJD

**FIGURE 4:**
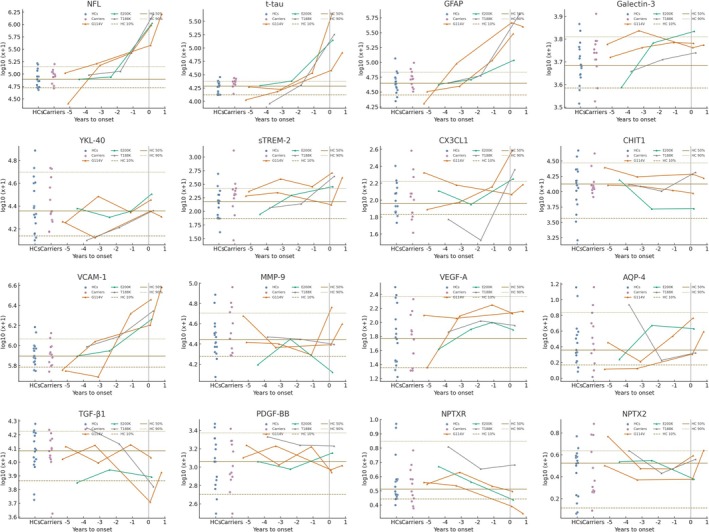
Plasma biomarker trajectories in preclinical CJD. Comparison of plasma biomarker levels at first sampling between 16 healthy family controls and 12 asymptomatic PRNP mutation carriers. Longitudinal changes are shown for four individuals who later developed symptomatic CJD. Serial data points from each individual are connected by simple linear interpolation, which may not represent the true biomarker trajectory between observed time points. Healthy control values and their major centiles are shown for reference. All biomarker data were log_10_(*x* + 1)‐transformed.

As summarized in Tables S8 and S9, a total of 16 asymptomatic *PRNP* mutation carriers were included in this study, among whom 4 eventually converted to symptomatic gCJD. The remaining 12 non‐converting carriers had a mean age of 36.1 years, including 6 males, with an estimated mean of 9.7 years to disease onset. Compared with 16 age‐ and sex‐matched non‐carrier family members, no significant differences in any biomarker levels were observed at baseline. During follow‐up, a total of 14 longitudinal plasma samples were collected (an average of 3.5 time points per individual). In the asymptomatic stage, mild elevations of NfL and GFAP were observed in two G114V carriers, and an increased level of VCAM‐1 in one carrier, each exceeding the 90th percentile of the control distribution. In contrast, all biomarkers remained stable in E200K and T188K carriers. After clinical onset, marked elevations in NfL, GFAP, t‐tau, and VCAM‐1 were observed (Figure 4). However, given the small sample size and limited longitudinal data, these findings should be interpreted with caution.

## 4 Discussion

This study provides a comprehensive plasma biomarker profile spanning the clinical spectrum of CJD, integrating markers of neuronal injury, glial activation, synaptic dysfunction, vascular damage, and neuroinflammation. Consistent with previous reports, symptomatic CJD was characterized by markedly elevated NfL, t‐tau, and GFAP levels, reflecting acute neuroaxonal degeneration and astrocytic activation, with strong group‐separating and discriminative performance. Concurrent increases in VCAM‐1 and decreases in TGF‐β1 further indicate significant vascular injury and BBB disruption. Higher NfL and t‐tau levels were associated with greater cognitive and functional impairment, underscoring their utility as indicators of disease severity and prognosis. However, in asymptomatic *PRNP* mutation carriers with genetic CJD, biomarker changes were minimal and appeared only in those with relatively slow‐progressing mutations, typically emerging close to clinical onset.

Consistent with prior reports, we found that plasma NfL and t‐tau are markedly elevated in symptomatic CJD, making them highly sensitive for group separation in this cohort (AUCs > 0.93 vs. HCs, and > 0.82 vs. FTD). This aligns with larger studies showing that blood NfL is significantly higher in prion disease compared to other neurodegenerative or rapidly progressive dementias, while plasma tau may provide somewhat greater specificity for prion disease [5, 6, 7]. We also observed strong correlations between these neuronal injury markers and clinical severity, consistent with their link to neuroaxonal damage. More recently, large‐scale CSF proteomics in sporadic CJD identified 201 differentially expressed proteins compared with controls and revealed partially overlapping yet distinguishable proteomic signatures across clinicopathological subtypes using UMAP clustering. Notably, the most upregulated proteins predominantly reflected neuroaxonal and cytoskeletal injury (e.g., MAPT and NFL), together with metabolic and signaling alterations (e.g., ENO2, RBKS, and CRKL), providing an independent proteomic context that is concordant with the neuronal injury signals captured by our plasma biomarkers [18]. GFAP was similarly elevated—reflecting astrocytic activation—but its discriminative performance is limited by its poor specificity, since many other neurological disorders also raise GFAP levels [19]. Although several biomarkers correlated with disease severity, only t‐tau remained an independent predictor of survival after adjusting for covariates. Previous studies similarly suggested that plasma NfL or GFAP provide limited additional prognostic value [19, 20]. Moreover, supportive care practices in CJD—such as enteral feeding—can markedly prolong survival [21, 22], potentially obscuring modest intrinsic associations between biomarker levels and disease duration.

This study evaluated plasma biomarkers associated with BBB integrity and cerebrovascular dysfunction, revealing significant vascular abnormalities in patients with CJD. VCAM‐1 is an adhesion molecule expressed on activated cerebral endothelial cells, facilitating the adhesion and transendothelial migration of peripheral immune cells [23]. Its soluble form is widely used as an indicator of endothelial activation and BBB leakage. In Alzheimer's disease (AD) and mild cognitive impairment, elevated VCAM‐1 levels have been associated with cognitive decline and hippocampal atrophy, and have even been incorporated into early blood biomarker panels [24, 25]. Our findings are consistent with previous postmortem studies demonstrating capillary damage and the loss of tight junction proteins in the CJD brain [4], supporting the role of VCAM‐1 as a peripheral indicator of neurovascular activation in CJD.

TGF‐β1, an anti‐inflammatory cytokine crucial for maintaining BBB integrity [26, 27], showed a marked decrease in CJD plasma, contrasting with its brain upregulation observed in prion models [28]. This likely reflects a collapse of anti‐inflammatory and BBB‐protective mechanisms during rapid neurodegeneration. Concurrently, reduced PDGF‐BB indicates impaired neurovascular support. Together, these vascular alterations suggest BBB dysfunction and endothelial inflammation in CJD, potentially driven by glial activation and proinflammatory cascades. Similar to early BBB breakdown seen in APOE4‐related AD [29], such vascular impairment may represent an early pathogenic rather than purely secondary event in prion disease.

Given the pronounced neuroinflammatory characteristics of prion diseases, we examined a panel of plasma biomarkers associated with glial activation. The results showed significant increases in CX3CL1 and sTREM2. CX3CL1, a chemokine secreted by neurons, regulates microglial activation through its receptor CX3CR1. Its elevation suggests active neuron–microglia signaling in CJD, consistent with observations in Parkinson's disease and dementia with Lewy bodies [30, 31], where CX3CL1 upregulation likely reflects a neuronal stress response. Galectin‐3, an important mediator of microglial activation and neuroinflammation in various neurodegenerative diseases, was markedly increased in terminal‐stage prion disease models, predominantly colocalizing with activated microglia and accompanied by upregulation of triggering receptor expressed on myeloid cells 2 and its downstream signaling pathways [32]. Although Galectin‐3 levels were not significantly elevated overall in our study, they showed a positive correlation with disease severity. Notably, Galectin‐3 levels were significantly lower in gCJD than in sCJD, suggesting a relatively attenuated microglial response in familial cases [33].

We also assessed synaptic function–related biomarkers NPTX2 and NPTXR, finding a significant decrease in plasma NPTXR levels in CJD patients. NPTX2/NPTXR, primarily released from functional excitatory synapses, serve as indicators of synaptic integrity, and their reduction reflects synaptic loss or dysfunction [34]. In line with reports of reduced CSF NPTX2 in AD and FTD associated with cognitive decline [35, 36, 37], the decrease of NPTXR in CJD suggests early synaptic degeneration driven by PrP^Sc^‐induced toxicity. Although blood‐based synaptic markers are not yet routinely used in CJD diagnostics, previous studies reporting elevated β‐synuclein and increased CSF SNAP‐25 and neurogranin levels support the presence of extensive synaptic damage [38, 39, 40]. Our findings extend this evidence, indicating that peripheral NPTXR reduction may serve as a feasible marker of synaptic pathology in prion disease.

We investigated whether peripheral biomarkers could detect imminent disease onset in asymptomatic *PRNP* mutation carriers. In our longitudinal cohort of 16 carriers (E200K, T188K, and G114V), plasma biomarkers remained stable during the asymptomatic phase, showing no significant differences from non‐carrier relatives. Over several years of follow‐up, biomarker trajectories were flat in non‐converting carriers, consistent with recent studies indicating that NfL and tau typically rise only within a few months before symptom onset [10]. Among the four converters, two G114V carriers showed mild pre‐symptomatic increases in NfL and GFAP approximately 1–2 years before clinical onset, and one also displayed a slight elevation in VCAM‐1. In contrast, E200K and T188K carriers exhibited abrupt biomarker surges only after symptom emergence. This pattern likely reflects differences in disease tempo: G114V represents a slower‐progressing form, allowing a longer prodromal phase with gradual biomarker elevation, whereas E200K and T188K follow a rapidly progressive CJD course with minimal preclinical window. These findings align with recent evidence that in slow mutations, GFAP and NfL rise 1–2 years before onset, while in fast mutations, they remain normal until just weeks prior to conversion. In our study, the pre‐symptomatic increases observed in G114V carriers were modest—only exceeding the 90th percentile of controls—and therefore not sufficient for predictive use. Overall, in most CJD subtypes, systemic biomarkers such as NfL, GFAP, and VCAM‐1 appear to change almost in parallel with symptom onset, while early detection remains best achieved through seed amplification assays like RT‐QuIC, which can identify PrP^Sc^ years before neurodegenerative markers become abnormal [10, 12].

Our study has several limitations. First, CJD diagnoses—especially for sporadic cases—were based on clinical and ancillary findings without autopsy confirmation, and we could not stratify analyses by molecular subtype, which may have introduced variability since different sCJD subtypes show distinct biomarker profiles. Second, the number of genetic CJD cases and converters was also small and heterogeneous, so conclusions about preclinical biomarker dynamics remain preliminary. Larger, mutation‐specific longitudinal studies are needed. Third, diagnostic analyses mainly compared CJD patients with healthy controls, which may overestimate specificity compared with real‐world scenarios involving other rapidly progressive dementias. Finally, prognostic evaluation was limited to baseline data, as most patients lacked longitudinal plasma sampling. Although we examined a broad biomarker panel, it was not exhaustive, and modest findings—especially for cytokines and synaptic proteins—require replication in independent cohorts.

In conclusion, our study shows that CJD pathology extends beyond neuronal loss, involving marked glial activation, synaptic dysfunction, and vascular injury—all reflected in blood biomarkers. Plasma NfL and t‐tau remain the most useful clinical markers, closely linked to disease severity, while elevated VCAM‐1 points to BBB disruption as a key feature. In asymptomatic *PRNP* mutation carriers, alterations in plasma biomarkers were generally subtle and became evident only in individuals with relatively slow‐progressing mutations as they approached clinical onset.

## Author Contributions

Li‐Yong Wu, Hong Ye, Zhong‐Yun Chen and Min Chu conceived and designed the study. Yi‐Hao Wang, Rui Liu, Jing Zhang, Ai‐ling Yue, Hua Lu, Qian‐qian‐He, Jia‐hui Hou and Yu‐fei Chen performed data acquisition, analysis, and interpretation. All authors contributed to drafting and critically revising the manuscript for important intellectual content. All authors read and approved the final version of the manuscript.

## Funding

“Yangfan 3.0” Diagnostic and Therapeutic Capability Enhancement Project of Beijing Municipal Hospital Administration (ZLRK202515); High‐Level Public Health Technical Talent Development Project (Talents Program 03‐03); Capital's Funds for Health Improvement and Research (2024‐2‐2018); National Natural Science Foundation of China (82271464, 81971011).

## Ethics Statement

The study was approved by the Ethics Committee of Xuanwu Hospital, Capital Medical University, China (Approval No. 2024‐180‐002), and was conducted in accordance with the Declaration of Helsinki.

## Consent

Written informed consent was obtained from all participants and/or their legal guardians.

## Conflicts of Interest

The authors declare no conflicts of interest.

## Supporting information


**Tables S1–S9:** cns70765‐sup‐0001‐TablesS1‐S9.docx.

## Data Availability

The data that support the findings of this study are available on request from the corresponding author. The data are not publicly available due to privacy or ethical restrictions.
