# Supplementary material for: Peripheral Signatures of Multidimensional Pathology in Symptomatic and Asymptomatic Creutzfeldt–Jakob Disease
Source: CNS Neurosci Ther. 2026 Jan 23;32(1):e70765. doi: 10.1002/cns.70765 (PMC12828275; doi:10.1002/cns.70765)
Supplement: Supplementary file 1 — Tables S1–S9: cns70765‐sup‐0001‐TablesS1‐S9.docx. [file CNS-32-e70765-s001.docx]

**Supplementary Table 1.** Differences in Biomarker Levels Between CJD and HCs

| **Biomarker** | **HCs(n=70)** | **CJD (n=130)** | **p_raw** | **p_age_corrected** | **p_FDR_corrected** |
| --- | --- | --- | --- | --- | --- |
| **Neuroinflammation** |  |  |  |  |  |
| Galectin-3 | 5987.00 (4610.00-7109.00) | 5785.00 (4472.00-6988.00) | 0.793 | 0.474 | 0.846 |
| YKL-40 | 24036.50 (17053.50-40400.25) | 25307.00 (17087.75-39882.00) | 0.856 | 0.593 | 0.856 |
| sTREM-2 | 130.02 (78.38-200.79) | 204.53 (109.28-216.86) | **0.008** | **0.025** | **0.017** |
| GFAP | 45922.02 (32883.47-68198.30) | 223683.85 (128614.48-362034.67) | **3.450E-39^#^** | **1.960E-34** | **2.760E-38** |
| CX3CL1 | 84.13 (53.25-129.72) | 119.13 (64.28-256.01) | **0.004** | **0.005** | **0.010** |
| CHIT1 | 12767.44 (8706.61-14267.89) | 12818.26 (6476.62-12818.26) | 0.209 | 0.633 | 0.298 |
| **BBB Integrity** |  |  |  |  |  |
| VCAM-1 | 787496.00 (640897.00-934972.00) | 1072603.00 (804598.75-1559921.00) | **8.531E-08** | **5.111E-10** | **3.413E-07** |
| MMP-9 | 32431.50 (22810.00-52896.00) | 44173.00 (19212.00-73933.00) | 0.519 | 0.301 | 0.593 |
| VEGF-A | 74.93 (27.68-157.48) | 53.63 (26.07-113.14) | 0.119**^#^** | 0.253 | 0.191 |
| Aquaporin-4 | 1.71 (0.74-3.86) | 1.43 (0.72-2.74) | 0.312 | 0.435 | 0.384 |
| TGF-β1 | 12201.19 (10123.27-14424.32) | 9659.69 (7068.54-11890.65) | **6.582E-6** | **1.486E-4** | **2.106E-4** |
| PDGF-BB | 1242.50 (617.43-2621.00) | 920.06 (405.32-1844.25) | **0.019^#^** | **0.033** | **0.033** |
| **Neuronal Injury** |  |  |  |  |  |
| NFL | 78267.52 (55841.43-101550.34) | 463943.20 (275613.72-838610.38) | **1.433E-49^#^** | **5.183E-44** | **2.292E-48** |
| t-tau | 22468.41 (18115.44-26641.89) | 144859.60 (66645.17-389684.15) | **8.319E-24** | **9.099E-30** | **4.437E-23** |
| **Synaptic Function** |  |  |  |  |  |
| NPTXR | 2.36 (1.82-3.01) | 2.01 (1.67-2.52) | **0.013** | **0.001** | **0.026** |
| NPTX2 | 2.40 (0.95-3.63) | 2.51 (1.38-4.08) | 0.224 | 0.116 | 0.298 |

^#^ Indicates that the data became normally distributed after log10(x + 1) transformation and were analyzed using a t-test; all other variables were analyzed using the Mann–Whitney U test.

**Abbreviation:** AQP-4, Aquaporin-4; BBB Integrity, Blood–Brain Barrier Integrity; CJD, Creutzfeldt-Jakob Disease; CX3CL1, C-X3-C Motif Chemokine Ligand 1; CHIT1, Chitotriosidase-1; Galectin-3, Galectin-3; GFAP, Glial Fibrillary Acidic Protein; MMP-9, Matrix Metalloproteinase-9; NFL, Neurofilament Light Chain; NPTX2, Neuronal Pentraxin 2; NPTXR, Neuronal Pentraxin Receptor; PDGF-BB, Platelet-Derived Growth Factor-BB; sTREM-2, Soluble Triggering Receptor Expressed on Myeloid Cells 2; t-tau, Total Tau Protein; TGF-β1, Transforming Growth Factor Beta 1; VCAM-1, Vascular Cell Adhesion Molecule-1; VEGF-A, Vascular Endothelial Growth Factor-A; YKL-40, Chitinase-3-Like Protein 1.

**Supplementary Table 2.** Differences in Biomarker Levels Between Sporadic and Genetic CJD

| **Biomarker** | **sCJD (n=111)** | **gCJD (n=19)** | **p_raw** | **p_age_corrected** | **p_FDR_corrected** |
| --- | --- | --- | --- | --- | --- |
| **Neuroinflammation** |  |  |  |  |  |
| Galectin-3 | 5886.00 (4668.00-7294.50) | 4396.00 (3968.00-5591.00) | **0.005^#^** | **0.034** | 0.087 |
| YKL-40 | 25524.00 (17124.50-43463.00) | 24663.00 (17082.00-35704.50) | 0.481**^#^** | 0.826 | 0.652 |
| sTREM-2 | 151.06 (90.57-275.93) | 117.18 (80.25-179.73) | 0.209 | 0.634 | 0.574 |
| GFAP | 232795.50 (133215.75-370510.25) | 148864.30 (103922.30-247050.60) | 0.060 | 0.267 | 0.322 |
| CX3CL1 | 129.72 (70.37-264.24) | 76.97 (49.50-144.97) | 0.128 | 0.307 | 0.511 |
| CHIT1 | 9540.99 (5935.96-17499.87) | 7440.01 (5561.30-15792.30) | 0.765 | 0.722 | 0.787 |
| **BBB Integrity** |  |  |  |  |  |
| VCAM-1 | 1072603.00 (829114.00-1689890.00) | 1036403.00 (635630.50-1283072.00) | 0.057 | 0.159 | 0.322 |
| MMP-9 | 43794.00 (19048.50-73304.00) | 44552.00 (21917.00-72733.50) | 0.787 | 0.433 | 0.787 |
| VEGF-A | 55.01 (28.64-109.38) | 44.77 (15.98-146.38) | 0.672**^#^** | 0.866 | 0.787 |
| Aquaporin-4 | 1.4 (0.71-2.63) | 1.24 (0.75-3.95) | 0.724 | 0.643 | 0.787 |
| TGF-β1 | 9265.67 (6817.18-11964.01) | 10487.16 (8657.62-12633.16) | 0.361 | 0.415 | 0.650 |
| PDGF-BB | 889.01 (417.73-1678.00) | 1678.00 (376.83-2117.00) | 0.396**^#^** | 0.335 | 0.650 |
| **Neuronal Injury** |  |  |  |  |  |
| NFL | 464414.70 (276830.15-876450.25) | 442038.80 (279866.60-644822.40) | 0.363**^#^** | 0.598 | 0.650 |
| t-tau | 171353.30 (64428.06-415954.45) | 119065.60 (69307.84-329850.30) | 0.406 | 0.724 | 0.650 |
| **Synaptic Function** |  |  |  |  |  |
| NPTXR | 2.01 (1.72-2.59) | 1.95 (1.54-2.36) | 0.489 | 0.463 | 0.652 |
| NPTX2 | 2.43 (1.40-3.87) | 4.31 (1.16-5.43) | 0.215 | 0.179 | 0.574 |

^#^ Indicates that the data became normally distributed after log10(x + 1) transformation and were analyzed using a t-test; all other variables were analyzed using the Mann–Whitney U test.

**Abbreviation:** AQP-4, Aquaporin-4; BBB Integrity, Blood–Brain Barrier Integrity; CJD, Creutzfeldt-Jakob Disease; CX3CL1, C-X3-C Motif Chemokine Ligand 1; CHIT1, Chitotriosidase-1; Galectin-3, Galectin-3; gCJD, genetic Creutzfeldt-Jakob Disease; GFAP, Glial Fibrillary Acidic Protein; MMP-9, Matrix Metalloproteinase-9; NFL, Neurofilament Light Chain; NPTX2, Neuronal Pentraxin 2; NPTXR, Neuronal Pentraxin Receptor; PDGF-BB, Platelet-Derived Growth Factor-BB; sCJD, sporadic Creutzfeldt-Jakob Disease; sTREM-2, Soluble Triggering Receptor Expressed on Myeloid Cells 2; t-tau, Total Tau Protein; TGF-β1, Transforming Growth Factor Beta 1; VCAM-1, Vascular Cell Adhesion Molecule-1; VEGF-A, Vascular Endothelial Growth Factor-A; YKL-40, Chitinase-3-Like Protein 1.

**Supplementary Table 3.** Differences in Biomarker Levels Between CJD and FTD

| **Biomarker** | **CJD (n=130)** | **FTD (n=145)** | **p_raw** | **p_age_corrected** | **p_FDR_corrected** |
| --- | --- | --- | --- | --- | --- |
| **Neuroinflammation** |  |  |  |  |  |
| Galectin-3 | 5785.00 (4472.00-6988.00) | 5687.00 (4957.00-7484.00) | 0.133 | 0.026 | 0.194 |
| YKL-40 | 25307.00 (17087.75-39882.00) | 31633.00 (19394.00-56218.00) | **0.046^#^** | 0.169 | 0.074 |
| sTREM-2 | 204.53 (109.28-216.86) | 197.00 (139.50-197.00) | 0.870 | 0.297 | 0.870 |
| GFAP | 223683.85 (128614.48-362034.67) | 61372.97 (42131.67-92466.97) | **2.673E-31^#^** | **6.685E-36** | **2.138E-30** |
| CX3CL1 | 119.13 (64.28-256.01) | 344.10 (170.81-598.56) | **8.229E-13** | **3.213E-12** | **3.292E-12** |
| CHIT1 | 12818.26 (6476.62-12818.26) | 13229.00 (9004.19-16327.31) | **0.003** | 0.119 | **0.007** |
| **BBB Integrity** |  |  |  |  |  |
| VCAM-1 | 1072603.00 (804598.75-1559921.00) | 919060.00 (747969.00-1191360.00) | **0.003** | **2.252E-05** | **0.006** |
| MMP-9 | 44173.00 (19212.00-73933.00) | 54743.00 (32153.00-103316.00) | **2.353E-04** | **1.912E-04** | **7.523E-04** |
| VEGF-A | 53.63 (26.07-113.14) | 63.11 (24.97-139.02) | 0.375**^#^** | 0.537 | 0.462 |
| Aquaporin-4 | 1.43 (0.72-2.74) | 1.75 (0.69-4.25) | 0.294 | 0.341 | 0.392 |
| TGF-β1 | 9659.69 (7068.54-11890.65) | 9815.74 (7736.89-11677.41) | 0.785 | 0.592 | 0.837 |
| PDGF-BB | 920.06 (405.32-1844.25) | 1,074.00 (471.01-1,992.00) | 0.408 | 0.714 | 0.467 |
| **Neuronal Injury** |  |  |  |  |  |
| NFL | 463943.20 (275613.72-838610.38) | 199461.44 (139,094.62-284563.49) | **5.224E-21^#^** | **1.621E-22** | **2.786E-20** |
| t-tau | 144859.60 (66645.17-389684.15) | 22925.24 (17946.79-28893.13) | **8.460E-35** | **1.003E-50** | **1.354E-33** |
| **Synaptic Function** |  |  |  |  |  |
| NPTXR | 2.01 (1.67-2.52) | 2.36 (1.90-2.94) | **0.003** | **0.002** | **0.006** |
| NPTX2 | 2.51 (1.37-4.10) | 2.04 (0.97-3.23) | **0.007** | **0.008** | **0.012** |

^#^ Indicates that the data became normally distributed after log transformation and were analyzed using a t-test; all other variables were analyzed using the Mann–Whitney U test.

**Abbreviation:** AQP-4, Aquaporin-4; BBB Integrity, Blood–Brain Barrier Integrity; CJD, Creutzfeldt-Jakob Disease; CX3CL1, C-X3-C Motif Chemokine Ligand 1; CHIT1, Chitotriosidase-1; FTD, frontotemporal dementia; Galectin-3, Galectin-3; GFAP, Glial Fibrillary Acidic Protein; MMP-9, Matrix Metalloproteinase-9; NFL, Neurofilament Light Chain; NPTX2, Neuronal Pentraxin 2; NPTXR, Neuronal Pentraxin Receptor; PDGF-BB, Platelet-Derived Growth Factor-BB; sTREM-2, Soluble Triggering Receptor Expressed on Myeloid Cells 2; t-tau, Total Tau Protein; TGF-β1, Transforming Growth Factor Beta 1; VCAM-1, Vascular Cell Adhesion Molecule-1; VEGF-A, Vascular Endothelial Growth Factor-A; YKL-40, Chitinase-3-Like Protein 1.

**Supplementary Table 4.**  Receiver Operating Characteristic (ROC) analysis of Biomarkers Between CJD and HCs

| **Biomarker** | **AUC (95% CI)** | **Cut-off** | **Sensitivity** | **Specificity** |
| --- | --- | --- | --- | --- |
| NFL | 0.976 (0.952 - 0.993) | 199506.20 | 0.900 | 0.971 |
| GFAP | 0.933 (0.900 - 0.964) | 86171.50 | 0.885 | 0.943 |
| t-tau | 0.932 (0.896 - 0.964) | 38618.95 | 0.854 | 0.929 |
| VCAM-1 | 0.730 (0.661 - 0.796) | 1036403.00 | 0.538 | 0.843 |
| TGF-β1 | 0.693^&^ (0.620 - 0.777) | 10052.30 | 0.600 | 0.771 |
| CX3CL1 | 0.624 (0.538 - 0.707) | 149.75 | 0.446 | 0.843 |
| sTREM-2 | 0.614 (0.524 - 0.698) | 206.13 | 0.500 | 0.800 |
| NPTXR | 0.607^&^ (0.529 - 0.687) | 2.44 | 0.738 | 0.486 |

^&^ Indicates reversed AUC (original AUC < 0.5).

**Abbreviation:** AUC, Area Under the Curve; CJD, Creutzfeldt-Jakob Disease; CX3CL1, C-X3-C Motif Chemokine Ligand 1; GFAP, Glial Fibrillary Acidic Protein; HCs, healthy controls; NFL, Neurofilament Light Chain; NPTXR, Neuronal Pentraxin Receptor; PDGF-BB, Platelet-Derived Growth Factor-BB; sTREM-2, Soluble Triggering Receptor Expressed on Myeloid Cells 2; t-tau, Total Tau Protein; TGF-β1, Transforming Growth Factor Beta 1; VCAM-1, Vascular Cell Adhesion Molecule-1.

**Supplementary Table 5.**  ROC Analysis of Biomarkers Between CJD and FTD

| **Biomarker** | **AUC (95% CI)** | **Cut-off** | **Sensitivity** | **Specificity** |
| --- | --- | --- | --- | --- |
| t-tau | 0.930 (0.893 - 0.962) | 52113.42 | 0.815 | 0.986 |
| GFAP | 0.874 (0.829 - 0.917) | 113545.50 | 0.815 | 0.828 |
| NFL | 0.819 (0.767 - 0.867) | 323147.60 | 0.708 | 0.814 |
| CX3CL1 | 0.750^&^ (0.691 - 0.807) | 230.76 | 0.738 | 0.676 |
| MMP-9 | 0.628^&^ (0.563 - 0.694) | 21665.00 | 0.323 | 0.903 |
| VCAM-1 | 0.605 (0.538 - 0.673) | 1412888.00 | 0.331 | 0.876 |
| NPTXR | 0.605^&^ (0.538 - 0.674) | 2.39 | 0.723 | 0.483 |
| CHIT1 | 0.602^&^ (0.529 - 0.678) | 12818.26 | 0.754 | 0.600 |

^&^ Indicates reversed AUC (original AUC < 0.5).

**Abbreviation:** AUC, Area Under the Curve; CJD, Creutzfeldt-Jakob Disease; CX3CL1, C-X3-C Motif Chemokine Ligand 1; FTD, frontotemporal dementia; GFAP, Glial Fibrillary Acidic Protein; MMP-9, Matrix metalloproteinase-9; NFL, Neurofilament Light Chain; NPTXR, Neuronal Pentraxin Receptor; t-tau, Total Tau Protein; VCAM-1, Vascular Cell Adhesion Molecule-1.

**Supplementary Table 6.**  Associations between plasma biomarkers and clinical or imaging measures in patients with CJD

| **Biomarker** | **Outcome** | **Partial_r** | **Partial_p value** | **Beta** | **SE** | **Reg_p value** | **FDR_corrected_p value** |
| --- | --- | --- | --- | --- | --- | --- | --- |
| Galectin-3 | MMSE | -0.234 | 0.007 | -0.001 | 3.65E-04 | 0.008 | 0.029 |
| Galectin-3 | MoCA | -0.178 | 0.043 | -0.001 | 2.61E-04 | 0.045 | 0.171 |
| Galectin-3 | MRC-PDRS | -0.293 | 0.001 | -0.001 | 2.79E-04 | 0.001 | 0.004 |
| YKL-40 | MMSE | -0.179 | 0.042 | -4.7E-05 | 2.33E-05 | 0.045 | 0.135 |
| YKL-40 | MRC-PDRS | -0.226 | 0.010 | -4.7E-05 | 1.79E-05 | 0.010 | 0.031 |
| GFAP | MRC-PDRS | -0.184 | 0.036 | -5E-06 | 2.39E-06 | 0.038 | 0.096 |
| VCAM-1 | MMSE | -0.242 | 0.006 | -2.3E-06 | 8.1E-07 | 0.006 | 0.029 |
| VCAM-1 | MoCA | -0.265 | 0.002 | -1.7E-06 | 5.69E-07 | 0.003 | 0.020 |
| VCAM-1 | MRC-PDRS | 0.264 | 0.002 | -1.9E-06 | 6.26E-07 | 0.003 | 0.010 |
| VCAM-1 | Quantification of DWI hyperintensity | -0.208 | 0.032 | 6.8E-07 | 3.18E-07 | 0.035 | 0.416 |
| NFL | MMSE | -0.293 | 0.001 | -3.4E-06 | 9.81E-07 | 0.001 | 0.006 |
| NFL | MoCA | -0.218 | 0.013 | -1.8E-06 | 7.07E-07 | 0.014 | 0.067 |
| NFL | MRC-PDRS | -0.318 | 2.26E-04 | -2.8E-06 | 7.56E-07 | 2.68E-04 | 0.002 |
| t-tau | MMSE | -0.308 | 3.59E-04 | -1.1E-05 | 2.93E-06 | 4.22E-04 | 0.006 |
| t-tau | MoCA | -0.263 | 0.003 | -6.4E-06 | 2.1E-06 | 0.003 | 0.020 |
| t-tau | MRC-PDRS | -0.431 | 3.00E-07 | -1.2E-05 | 2.16E-06 | 4.12E-07 | 4.79E-06 |
| t-tau | Disease duration | -0.242 | 0.011 | -1.40E-04 | 5.65E-05 | 0.012 | 0.031 |

**Abbreviation:** Creutzfeldt-Jakob Disease; Galectin-3, Galectin-3; GFAP, Glial Fibrillary Acidic Protein; NFL, Neurofilament Light Chain; t-tau, Total Tau Protein; VCAM-1, Vascular Cell Adhesion Molecule-1; YKL-40, Chitinase-3-Like Protein 1.

**Supplementary Table 7.**  Associations of plasma biomarkers levels with survival time

| Plasma biomarker | Univariate Cox regression | |  | Multivariate Cox regression* | |
| --- | --- | --- | --- | --- | --- |
|  | HR (95% CI) | p-value |  | HR (95% CI) | p-value |
| Galectin-3 | 1.082 (0.278–4.210) | 0.909 |  | 0.813 (0.197–3.360) | 0.775 |
| YKL-40 | 1.201 (0.649–2.221) | 0.560 |  | 0.978 (0.500–1.913) | 0.949 |
| sTREM-2 | 1.122 (0.611–2.058) | 0.711 |  | 1.117 (0.607–2.054) | 0.7219 |
| GFAP | 1.238 (0.785–1.953) | 0.359 |  | 1.581 (0.921–2.712) | 0.097 |
| CX3CL1 | 1.154 (0.706–1.884) | 0.568 |  | 0.903 (0.543–1.500) | 0.692 |
| CHIT1 | 0.612 (0.331–1.133) | 0.11 |  | 0.592 (0.315–1.112) | 0.103 |
| VCAM-1 | 1.589 (0.647–3.905) | 0.312 |  | 1.326 (0.525–3.350) | 0.551 |
| MMP-9 | 1.093 (0.646–1.851) | 0.740 |  | 0.931 (0.539–1.607) | 0.796 |
| VEGF-A | 1.020 (0.664–1.566) | 0.928 |  | 0.814 (0.510–1.297) | 0.386 |
| Aquaporin-4 | 1.270 (0.633–2.549) | 0.501 |  | 0.882 (0.435–1.789) | 0.729 |
| TGF-β1 | 1.053 (0.380–2.918) | 0.922 |  | 0.946 (0.361–2.479) | 0.911 |
| PDGF-BB | 1.822 (1.029–3.228) | 0.040 |  | 1.628 (0.887–2.989) | 0.116 |
| NFL | 1.986 (1.296–3.042) | 0.002 |  | 1.729 (1.102–2.712) | **0.017** |
| t-tau | 0.995 (0.643–1.539) | 0.982 |  | 0.885 (0.562–1.391) | 0.595 |
| NPTXR | 0.352 (0.073–1.699) | 0.196 |  | 0.264 (0.051–1.373) | 0.113 |
| NPTX2 | 2.321 (0.904–5.963) | 0.080 |  | 2.272 (0.898–5.751) | 0.083 |

^*^All multivariate Cox regression analyses included sex, age at blood sample and time from onset to sample collection as covariates

**Abbreviation:** AQP-4, Aquaporin-4; BBB Integrity, Blood–Brain Barrier Integrity; CJD, Creutzfeldt-Jakob Disease; CX3CL1, C-X3-C Motif Chemokine Ligand 1; CHIT1, Chitotriosidase-1; Galectin-3, Galectin-3; gCJD, genetic Creutzfeldt-Jakob Disease; GFAP, Glial Fibrillary Acidic Protein; MMP-9, Matrix Metalloproteinase-9; NFL, Neurofilament Light Chain; NPTX2, Neuronal Pentraxin 2; NPTXR, Neuronal Pentraxin Receptor; PDGF-BB, Platelet-Derived Growth Factor-BB; sCJD, sporadic Creutzfeldt-Jakob Disease; sTREM-2, Soluble Triggering Receptor Expressed on Myeloid Cells 2; t-tau, Total Tau Protein; TGF-β1, Transforming Growth Factor Beta 1; VCAM-1, Vascular Cell Adhesion Molecule-1; VEGF-A, Vascular Endothelial Growth Factor-A; YKL-40, Chitinase-3-Like Protein 1.

**Supplementary Table 8.** Demographic and Clinical Characteristics of Patients with Preclinical CJD and Family-Matched Controls

|  | Preclinical gCJD (n=12) | Family-matched control (n=16) | p-value |
| --- | --- | --- | --- |
| Age | 36.1±8.5 | 37.6±9.4 | 0.672 |
| gender(M/F) | 6/6 | 7/9 |  |
| MMSE | 29.3±0.8 | 29.1±1.1 | 0.481 |
| MoCA | 28.7±1.2 | 28.1±1.6 | 0.525 |
| Years from expected onset age | 9.7±7.4 | - | - |

**Abbreviation:** CJD, Creutzfeldt-Jakob Disease; M, male; F, female; MMSE, Mini-Mental State Examination; MoCA, Montreal Cognitive Assessment

**Supplementary Table 9.** Demographic Data of Four Cases Who Eventually Developed Symptomatic gCJD

|  | Case 1 | Case 2 | Case 3 | Case 4 |
| --- | --- | --- | --- | --- |
| Baseline age | 40s | 20s | 50s | 40s |
| Sex | Female | Male | Male | Male |
| Mutation site | G114V | G114V | T188K | E200K |
| Initial symptom | Extrapyramidal symptoms | Cognitive impairment | Gait disorder | Cognitive impairment |
| Clinical features at presentation, n (%) | | | | |
| Cognitive | + | + | + | + |
| Psychiatric | - | - | + | - |
| Visual | - | - | - |  |
| Extrapyramidal | + | - | - | - |
| Pyramidal | - | - | - | - |
| Cerebellar | + | - | + | - |
| Myoclonus | - | - | + | - |
| Mutism | - | - | - | - |
| CSF 14-3-3 positive | + | + | + | - |
| Periodic discharges on EEG | - | - | + | - |
| Hyperintensity on DWI | + | + | + | + |
| Positive RT-QuIC | + | + | + | + |
| Codon 129 genotype | MM | MM | MM | MM |

**Abbreviation:** CJD,Creutzfeldt-Jakob disease; DWI, Diffusion Weighted Imaging; EEG, electroencephalogram; MM,methionine homozygosity; RT-QuIC, Real-time qualing-induced conversion.
